# The complete chloroplast genome sequence of *Echinops davuricus* Trevir. (Asteraceae), a medicinal plant

**DOI:** 10.1080/23802359.2026.2715802

**Published:** 2026-08-14

**Authors:** Junjie Wei, Peiyan Ai, Xiaoming Guo, Tiankun Wu, Zhenbei Wu, Chunhui Li, Peng Hu, Bo Zhang, Leyuan Li, Jianfeng Chang, Chengxue Pan, Lianzhen Li

**Affiliations:** a College of Agronomy, Henan Agricultural University, Zhengzhou, China; b College of Life Sciences, Henan Agricultural University, Zhengzhou, China; c Department of Pharmacy, The Fifth Clinical Medical College of Henan University of Chinese Medicine (Zhengzhou People’s Hospital), Zhengzhou, China; d School of Pharmaceutical Sciences, Zhengzhou University, Zhengzhou, China

**Keywords:** Echinops davuricus, chloroplast genome, phylogenetic analysis

## Abstract

*Echinops davuricus* Trevir. (Asteraceae) is a perennial medicinal herb. In this study, we sequenced and assembled its complete chloroplast genome. The plastome spans 153,168 bp with an overall GC content of 37.76%, showing a typical quadripartite structure. It encodes 131 genes in total, including 87 protein-coding genes (PCGs), 36 transfer RNA (tRNA) genes and eight ribosomal RNA (rRNA) genes. Phylogenetic analysis based on whole plastomes strongly supports the monophyly of both Echinops and Cardueae. This work enriches genomic resources for Echinops and provides a valuable basis for its phylogenetic and evolutionary studies.

## Introduction

*Echinops davuricus* Trevir. 1819, a member of the Cardueae tribe in the Asteraceae (Compositae), is an erect perennial herb, distinguished by bipinnatipartite leaves with entire or sparsely spiny-toothed lobes. Each plant typically bears 1–3 spherical inflorescences containing multiple single-flowered capitula with blue corollas. *E. davuricus* distributes in temperate regions. Its native range extends from southern Siberia to Korea. In China, this species is mainly distributed in Gansu, Hebei, Heilongjiang, Henan, Jilin, Liaoning, Inner Mongolia, Ningxia, Shaanxi, Shandong, and Shanxi provinces (Shi et al. 2011). The roots of *E. davuricus* are used in traditional Chinese medicine under the name ‘Echinopsis Radix’ or ‘Yuzhou Loulu,’ and are reputed to clear heat, remove toxin, disperse abscesses, promote lactation, relax sinews and unblock the meridians (Chinese Pharmacopoeia Commission 2022). Phytochemical studies have demonstrated that this species contains thiophenes, flavonoids, phenolic compounds, sesquiterpenoids, and other compounds (Olennikov and Chirikova 2024; Sun et al. 2024b). Pharmacological investigations have further revealed a range of bioactivities, including antitumor, anti-inflammatory, hepatoprotective, insecticidal and parasiticidal, antibacterial, and antiviral effects (Sun et al. 2024a, 2024b).

Despite its significant medicinal value, genomic information for this species remains scarce. Chloroplast genomes are widely used in studies of population genetics, phylogenetics, taxonomy, plant genetic conservation, and DNA barcoding (Jansen et al. 2005; Jansen and Ruhlman 2012). To date, the chloroplast genome of *E. davuricus* has not been reported. According to the *Flora of China*, the genus *Echinops* comprises approximately 120 species distributed across Africa, Asia, and Europe, with 17 species (five endemic) occurring in China (Shi et al. 2011); however, chloroplast genomes have been published for a limited number of species in this genus to date (Abdullah et al. 2026). In this study, we assembled and annotated the complete chloroplast genome of *E. davuricus* and constructed a phylogenetic tree including representative species of the tribe Cardueae within Asteraceae. These findings provide valuable resources for future species identification, phylogenetic analysis, and the development and utilization of medicinal plant resources.

## Materials and methods

**Figure 1.**
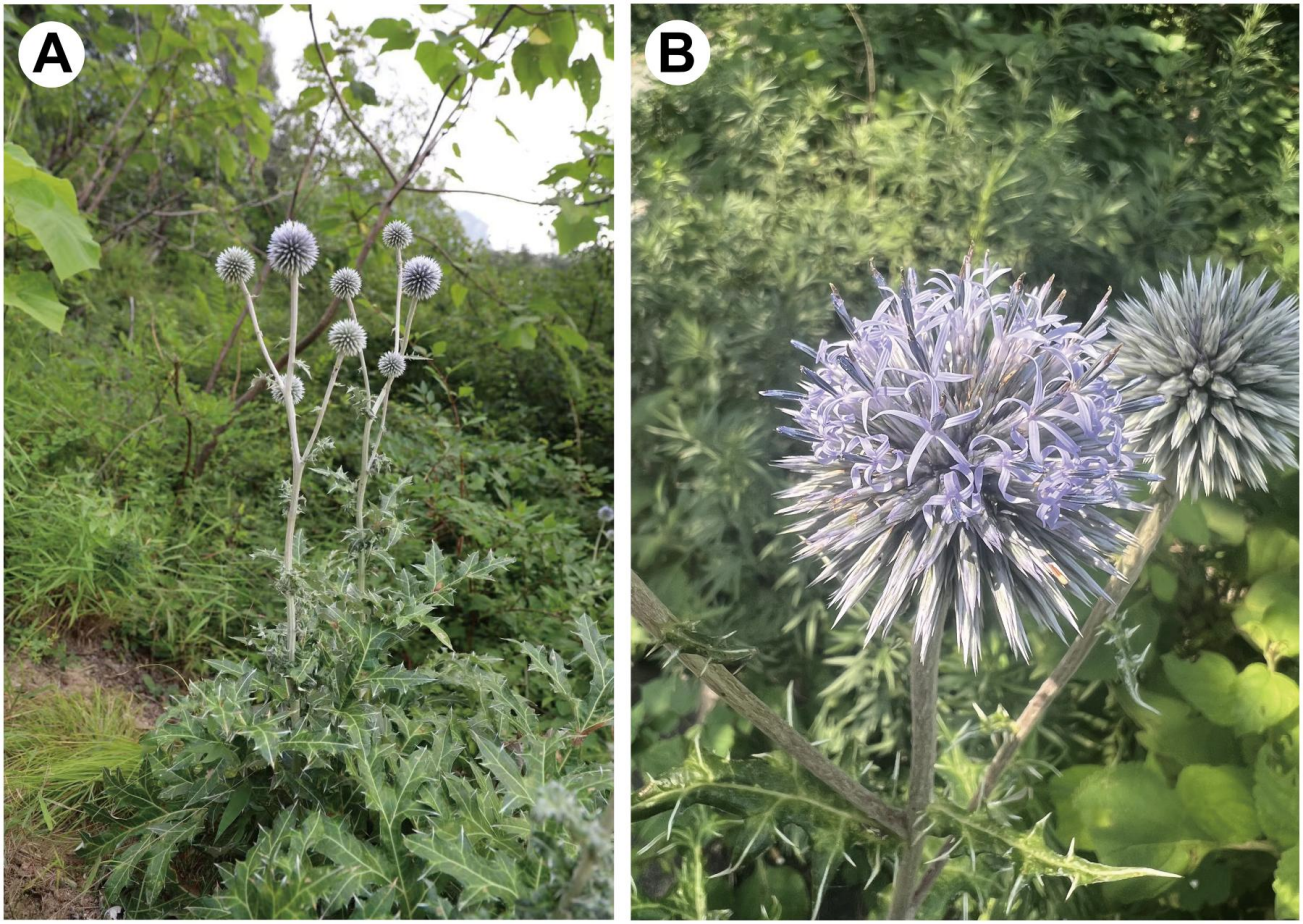
Morphology of *Echinops davuricus*. (A) Whole plant. (B) A flowering spherical inflorescence with blue corollas. Photographs taken by Chengxue Pan at Wanxian Mountain, Huixian, China..

Fresh leaves were collected at Wanxian Mountain, Huixian, Xinxiang, China (35.7108 N, 113.5983 E). The specimen was identified as *Echinops davuricus* Trevir. by Prof. Chengxue Pan. A voucher specimen (PC20250701001) was deposited in the Herbarium of School of Pharmaceutical Sciences, Zhengzhou University (contact: Chengxue Pan, pancx@zzu.edu.cn; Figure 1).

Total genomic DNA was extracted using the CTAB method (Doyle and Doyle 1987). Sequencing libraries were constructed using Hieff NGS^®^ OnePot Pro DNA Library Prep Kit V4 (Yeasen Biotechnology, Shanghai, China; Cat. No. 12972) according to the manufacturer’s protocol, and paired-end (PE150) sequencing was performed on the DNBSEQ-T7 platform by Bena Technology Co., Ltd. (Wuhan, China). Raw data was firstly filtered by fastp v1.3.4. The chloroplast genome was assembled using GetOrganelle v1.7.7.1 (Jin et al. 2020) and visualized with Bandage v0.8.1 (Wick et al. 2015). Clean reads were mapped to the chloroplast reference genome using Bowtie2 v2.4.4, and the resulting plastidial reads were quantified and summarized using Samtools v1.13. Genome annotation was conducted using PGA (Qu et al. 2019) with *Ainsliaea henryi* (NC_086780.1) as the reference, followed by manual curation in Geneious Prime v2025.0.2 (Kearse et al. 2012). A circular chloroplast genome map was generated using OGDraw (Greiner et al. 2019).

For phylogenetic analysis, complete chloroplast genome sequences of *E. davuricus* and other 39 species covering nearly all available genera within tribe Cardueae (Asteraceae), together with the sequences of four outgroup species, were used to construct phylogenetic tree (Table S1). Multiple sequence alignment was performed using MAFFT v7.505 (Katoh et al. 2002) with the ‘–auto’ strategy and then TrimAI v1.2 was used to trim ambiguous alignments (Capella-Gutiérrez et al. 2009). The orientations of the small single-copy (SSC) and large single-copy (LSC) regions were adjusted to be consistent with *E. davuricus* (Hu et al. 2025). A maximum-likelihood (ML) phylogenetic tree was reconstructed using IQ-TREE implemented in PhyloSuite v1.2.2 (Zhang et al. 2020) under the best-fit model TVM+F + I + I + R5, with 1,000 bootstrap replicates. *Cichorium intybus*, *Takhtajaniantha austriaca*, *Tragopogon dubius*, and *Youngia denticulata* (tribe Cichorieae, Asteraceae) were designated as outgroup.

## Results

Paired-end sequencing of *Echinops davuricus* generated 9.638 GB of data (64,253,802 reads in total). The overall Q20 rate reached 99.09% and the Q30 rate was 96.74%, demonstrating high sequencing quality. After quality filtering with fastp, 64,150,004 high-quality clean reads were retained. Subsequent mapping against the *E. davuricus* chloroplast genome (GenBank: PZ166814.1) *via* Bowtie2 recovered 860,451 plastid-derived reads, accounting for 1.34% of all clean reads. The chloroplast genome is 153,168 bp in length and exhibits a typical quadripartite structure, comprising a large single-copy (LSC) region of 84,304 bp, a small single-copy (SSC) region of 18,470 bp, and a pair of inverted repeat (IR) regions of 25,197 bp each. The overall GC content is 37.76%, with corresponding values of 35.90% in the LSC, 31.50% in the SSC, and 43.14% in the IR regions. Sequencing depth ranged from 177× to 20,005×, with an average coverage of 863× (Figure S1).

**Figure 2.**
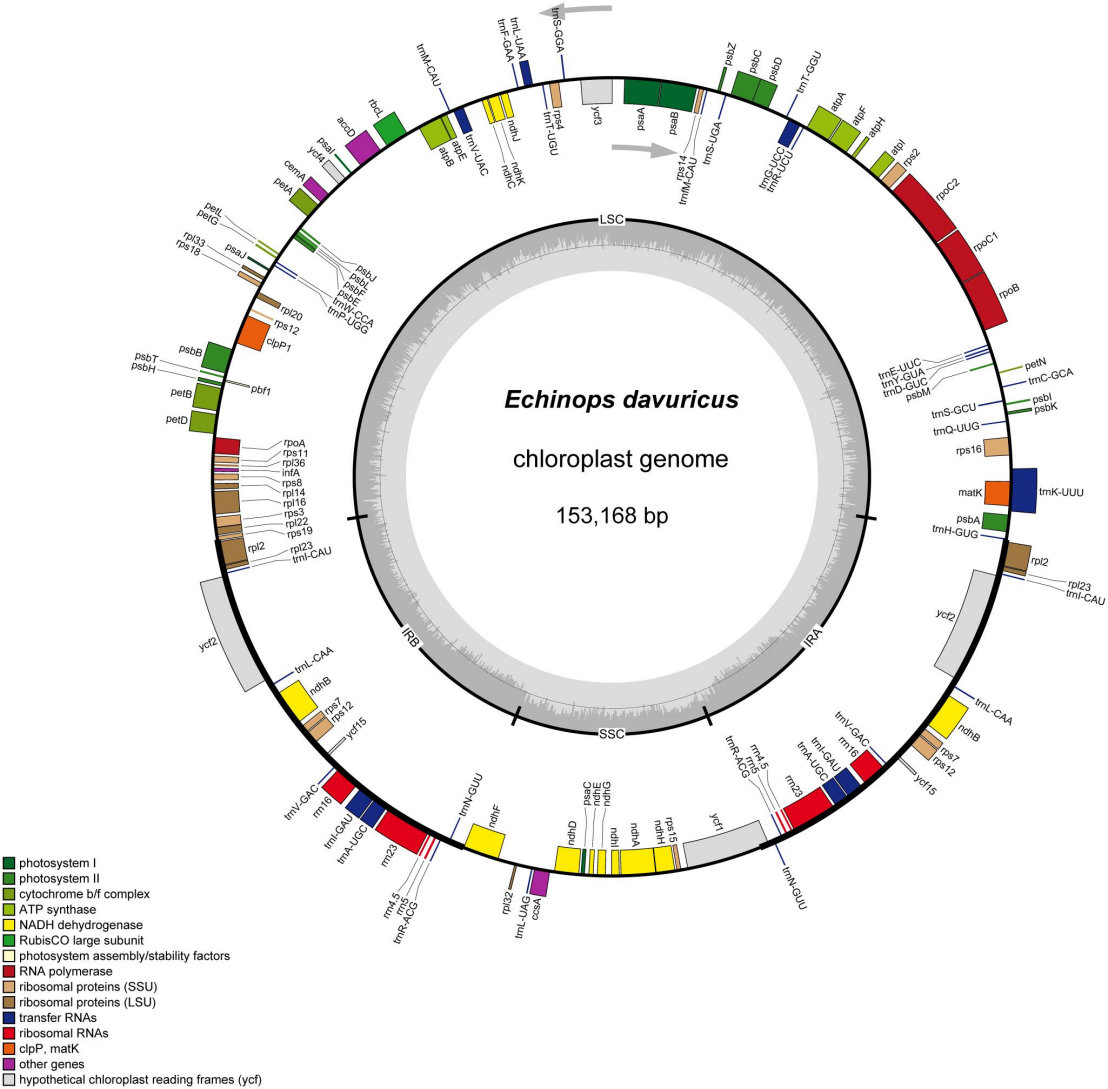
The complete chloroplast genome map of *Echinops davuricus.* The outer circle shows the distribution of genes. Genes inside the circle are transcribed in a clockwise direction, whereas those outside the circle are transcribed in a counterclockwise direction. Genes are color-coded according to their functional categories. In the inner circle, GC content is displayed in dark gray shade, while AT content is displayed in light gray shade.

The genome comprises 131 genes, including 87 protein-coding genes (PCGs), 36 transfer RNA (tRNA) genes, and eight ribosomal RNA (rRNA) genes (Figure 2). Among these, 18 genes are duplicated in the IR regions, including seven PCGs (*ndhB, rpl2, rpl23, rps7, rps12, ycf2,* and *ycf15*), seven tRNA genes (*trnA-UGC, trnI-CAU, trnI-GAU, trnL-CAA, trnN-GUU, trnR-ACG,* and *trnV-GAC*), and four rRNA genes (*rrn5, rrn16, rrn4.5,* and *rrn23*). Three genes (*rps12, clpP,* and *ycf3*) contain two introns each. In addition, nine PCGs (*ndhA, ndhB, petB, petD, atpF, rpl2, rpl16, rps16,* and *rpoC1*) and six tRNA genes (*trnA-UGC, trnG-UCC, trnI-GAU, trnK-UUU, trnL-UAA,* and *trnV-UAC*) each harbor a single intron. Furthermore, eleven genes are cis-spliced (*rps16, atpF, rpoC1, ycf3, clpP, petB, petD, rpl16, rpl2, ndhB,* and *ndhA*; Figure S2), whereas one gene (*rps12*) is trans-spliced (Figure S3).

**Figure 3.**
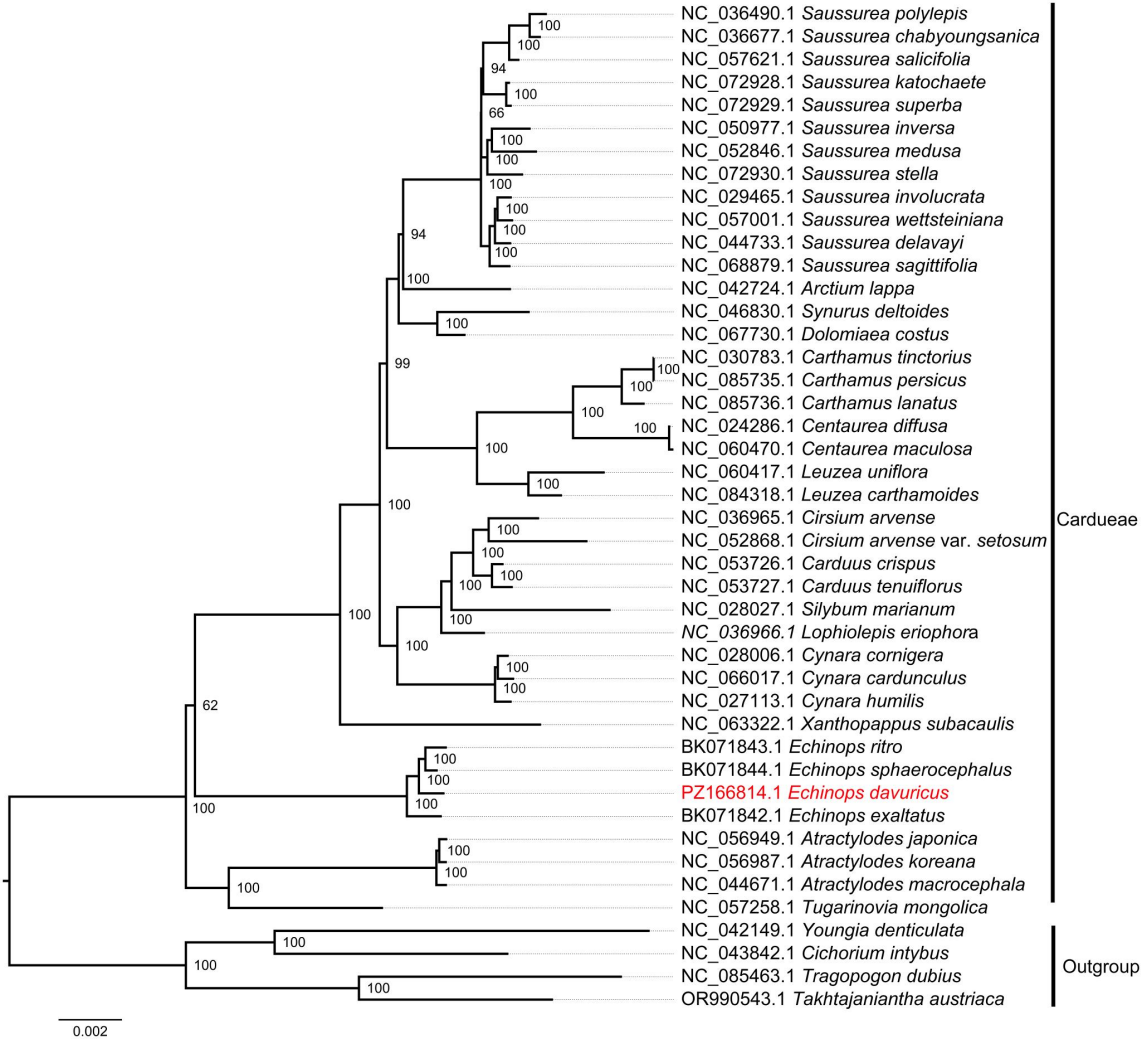
Phylogenetic relationships of *Echinops davuricus* and other species under cardueae. The tree, with aligned tip labels (indicated by dash-dotted guide lines), was constructed using the maximum likelihood (ML) method with 1,000 bootstrap replicates. Bootstrap values are shown around the branches. *Arctium lappa* (NC_042724.1), *Atractylodes japonica* (NC_056949.1, Shi et al. 2021), *Atractylodes koreana* (NC_056987.1, Xie et al. 2021), *Atractylodes macrocephala* (NC_044671.1, Li and Yang 2020), *Carduus crispus* (NC_053726.1, Jung et al. 2021), *Carduus tenuiflorus* (NC_053727.1, Jung et al. 2021), *Carthamus lanatus* (NC_085736.1, Yang et al. 2023), *Carthamus persicus* (NC_085735.1, Yang et al. 2023), *Carthamus tinctorius* (NC_030783.1), *Centaurea diffusa* (NC_024286.1), *Centaurea maculosa* (NC_060470.1, Park et al. 2019), *Cirsium arvense* (NC_036965.1), *Cirsium arvense* var. *setosum* (NC_052868.1, Xie et al. 2019), *Cynara cardunculus* (NC_066017.1), *Cynara cornigera* (NC_028006.1), *Cynara humilis* (NC_027113.1, Curci and Sonnante 2016), *Dolomiaea costus* (NC_067730.1), *Echinops davuricus* (PZ166814.1, this study), *Echinops exaltatus* (BK071842.1, Abdullah et al. 2026), *Echinops ritro* (BK071843.1, Abdullah et al. 2026), *Echinops sphaerocephalus* (BK071844.1, Abdullah et al. 2026), *Leuzea carthamoides* (NC_084318.1), *Leuzea uniflora* (NC_060417.1), *Lophiolepis eriophora* (NC_036966.1), *Saussurea chabyoungsanica* (NC_036677.1, Cheon et al. 2017), *Saussurea delavayi* (NC_044733.1), *Saussurea inversa* (NC_050977.1, Wang et al. 2021), *Saussurea involucrata* (NC_029465.1), *Saussurea katochaete* (NC_072928.1, He et al. 2023), *Saussurea medusa* (NC_052846.1, Wang et al. 2020), *Saussurea polylepis* (NC_036490.1, Yun et al. 2017), *Saussurea sagittifolia* (NC_068879.1, Liu et al. 2023), *Saussurea salicifolia* (NC_057621.1, Chen et al. 2019), *Saussurea stella* (NC_072930.1, He et al. 2023), *Saussurea superba* (NC_072929.1, He et al. 2023), *Saussurea wettsteiniana* (NC_057001.1, Luo et al. 2021), *Silybum marianum* (NC_028027.1, Shim 2020), *Synurus deltoides* (NC_046830.1), *Tugarinovia mongolica* (NC_057258.1, Cong and Jiang 2022), *Xanthopappus subacaulis* (NC_063322.1, Ma et al. 2022), *Cichorium intybus* (NC_043842.1, Yang et al. 2019), *Takhtajaniantha austriaca* (OR990543.1), *Tragopogon dubius* (NC_085463.1, Huang et al. 2024), *Youngia denticulata* (NC_042149.1, Do et al. 2019).

Phylogenetic analysis strongly supported the monophyly of Cardueae, with all sampled species forming a well-supported clade. Within the genus *Echinops*, the topology indicates that *E. exaltatus* and *E. davuricus* diverged earlier than the *E. ritro* – *E. sphaerocephalus* pair, with all nodes receiving maximum bootstrap support (Figure 3).

## Discussion and conclusion

This study reports the complete chloroplast genome of *E. davuricus*. The genome is 153,168 bp in length and comprises 131 predicted genes, exhibiting a typical quadripartite structure. These characteristics are highly similar to those of other *Echinops* species (Abdullah et al. 2026; Table S1), indicating a high level of conservation in plastid genomes within the genus.

Our plastome-based phylogenetic analysis further confirmed that the tribe Cardueae, to which *Echinops* belongs, is monophyletic, consistent with Abdullah et al. (2026) and Herrando-Moraira et al. (2019). Herrando-Moraira et al. (2019) reconstructed the phylogeny of 76 representative species of Cardueae using nuclear and plastid DNA and proposed a classification comprising 12 subtribes. Their plastid-based analysis, based on 87 chloroplast genes, included eight genera overlapping with this study, and the inferred relationships are largely congruent with our results. Similarly, Abdullah et al. (2026), using complete plastome sequences for 76 species across 51 genera, recovered phylogenetic patterns largely consistent with ours, with only minor discrepancies observed within Centaureinae (only *Centaurea* and *Carthamus* are present in this study). Within *Echinops*, consistent with Abdullah et al. (2026), all *Echinops* species formed a monophyletic group. However, our phylogeny further clarifies that *E. davuricus* occupies an intermediate position, branching off after *E. exaltatus* but prior to the divergence of the *E. ritro*–*E. sphaerocephalus* sister pair.

In summary, this study successfully assembled and annotated the chloroplast genome of *E. davuricus* and clarified its phylogenetic position within *Echinops* and Cardueae, thereby enriching available genomic resources and providing further insights into plastome evolution and phylogenetic relationships within the genus.

## Supplementary Material

Supplemental Material

## Data Availability

The genome sequence data that support the findings of this study are openly available in GenBank of NCBI at https://www.ncbi.nlm.nih.gov/ under accession no. PZ166814.1. The associated BioProject, Bio-Sample and SRA numbers are PRJNA1445089, SAMN56777926 and SRR37846525, respectively.
