# Supplementary material for: The complete chloroplast genome sequence of Echinops davuricus Trevir. (Asteraceae), a medicinal plant
Source: Mitochondrial DNA B Resour. 2026 Aug 14;11(9):1042–7. doi: 10.1080/23802359.2026.2715802 (PMC13479520; doi:10.1080/23802359.2026.2715802)
Supplement: Supplementary file 1 [file TMDN_A_2715802_SM6873.pdf]

GENOME ANNOUNCEMENT SUBMISSION CHECKLIST (version: 20230601)

Incomplete submissions will not move on into the peer-review process until these requirements are met. Authors may submit their paper in any scholarly format or layout. Although there are no strict formatting requirements, all manuscripts must contain the essential elements needed to evaluate a manuscript, below is a check list for your convenience. However, this checklist does not preclude reading the information for authors. (https://www.tandfonline.com/action/authorSubmission?journalCode=tmdn20&page=instructions#ffs)

- ☑ Is the abstract less than 200 words?
- ☑ Is the text less than 1200 words (Not including Abstract)?
- ☑ Is the main text divided into "Introduction, Materials and Methods, Results, Discussion and Conclusion" sections?
- ☑ Are the supplementary figures and tables mentioned and described in the main text? The coverage-depth map is required for all studies using high-throughput DNA sequencing technologies. The PCR primers must be provided for studies using PCR amplification and Sanger methods.
- ☑ Have you included 3-5 keywords describing your paper? The keywords should not duplicate the words already used in the title.
- ☑ Have I included the following mandatory figures, a maximum of three figures are allowed in the main text :
  - ☑ Species Reference Image, please provide the image source in the respective footnote/legends and clarify whether the photo of the species was taken by the authors. If the picture was taken from a previously published article, please include **a reference to the original source**, and state whether permission was obtained to include them in your article from the copyright holder (if applicable).
  - ☑ Genome Feature Map.
  - ☑ Phylogenetics Tree, the database accession numbers, and their citations should be provided in the figure legend.
  - ☑ Read coverage plot for studies using high throughput DNA sequencing technologies or PCR gel plot for studies using PCR amplification. This should be included in the supplementary materials.
  - ☑ Maps showing the structure of complex genes, such as cis-splicing and trans-splicing genes, if applicable. This should be included in the supplementary materials.
- ☑ Are the figures arranged in the same order as they are mentioned in the manuscript?
- ☐ All items in the figure must be legible, to ensure reviewers are able to read the article at 100% magnification, therefore figures must satisfy the following image quality requirements:
  - ☑ Image resolution should be at least 600 dpi;
  - ☑ font size should at least 8 for font type Arial;
  - ☑ width of the image around 6 inches;
  - ☑ The blank space, including the line spacing, in the figure should be minimized.
  - ☑ No text should overlap with the lines.
- ☑ Do the figure legends contain sufficient information for the users to understand the meaning of the figures? The legend must be detailed enough for the figure to be understandable without referring to the text. ALL features visible in the figure must be explained
- ☑ Is the scientific name of the species correct?
- ☑ Is the name of the species in full and the authority included the first time the species is mentioned (e.g. __Dicentrarchus labrax__ Linaeus 1758)?
- ☑ Is the information on the specimen deposit included?

[The deposition of the specimen and DNA is required.]

☑ Is the name of the herbarium/museum or other institution included?
[Please give details on where the specimen or its DNA is stored, including its accession/voucher number. Specimens must be stored in a tissue collection with a museum or herbarium.]

☑ Is the voucher number included?

☑ Are the email contacts of the people in charge of the collection included?
[suggested text: "A specimen was deposited at -institution name- (URL, contact person and email) under the voucher number XXXXXX"]

☑ Are GenBank/ENA/DDBJ and BioProject, SRA, and Bio-Sample accession numbers of the genome included in the "Data availability statement"?

It is mandatory that authors provide a data availability statement (DAS), detailing where data associated with a paper can be found and how it can be accessed. Data must be deposited in a [recognized data repository](http://authorservices.taylorandfrancis.com/data-repositories/) prior to submission. We suggest the following template: "The genome sequence data that support the findings of this study are openly available in GenBank of NCBI at [https://www.ncbi.nlm.nih.gov] (https://www.ncbi.nlm.nih.gov/) under the accession no. XXXXXXX-XXXXXX. The associated BioProject, SRA, and Bio-Sample numbers are PRJNXXXXX, SXXXXXX, and SXXXXX respectively."

☑ Are GenBank/ENA/DDBJ and BioProject, SRA, and Bio-Sample accession numbers activated, i.e. public?.
[These must be activated and live at submission stage. Do not submit a manuscript with pending or temporary GenBank accession numbers or your manuscript will be rejected.]

☑ Is the specimen collection site properly identified with geographic coordinates (e.g. latitude 38.8951 and longitude -77.0364).
[The locality where the specimen was collected is required. Please give the exact location of where the specimen was collected, preferably with geospatial coordinates.]

☑ Acknowledgment of financial support, including grant numbers. Please supply all details required by your funding and grant-awarding bodies as follows:

- For single agency grants use: "This work was supported by the [Funding Agency] under Grant [number xxxx].". For multiple agency grants use:

- "This work was supported by the [Funding Agency #1] under Grant [number xxxx]; [Funding Agency #2] under Grant [number xxxx]; and [Funding Agency #3] under Grant [number xxxx]."

☑ Disclosure statement. This is to acknowledge any financial interest or benefit that has arisen from the direct applications of your research. [Further guidance on what is a conflict of interest and how to disclose it](http://authorservices.taylorandfrancis.com/what-is-a-conflict-ofinterest/).

☑ Declaration of interest included. [Each Mitogenome Announcement requires a Declaration of Interest (https://authorservices.taylorandfrancis.com/what-is-a-conflict-ofinterest/).]

☑ Author Contributions statement included? Please provide an author contributions statement at the end of your article, before the references, that outlines which author(s) were involved in the conception and design, or analysis and interpretation of the data; the drafting of the paper, revising it critically for intellectual content; and the final approval of the version to be published; and that all authors agree to be accountable for all aspects of the work. Please use author initials for contributions to ensure conciseness e.g.

"AB and CD conceived the project, CD and EF conducted experiments, AB, CD and EF wrote the manuscript"

☑ All original research papers involving humans, animals, plants, biological material, protected or non-public datasets, collections or sites, must include a written statement confirming that all the relevant permissions, licenses and/or ethical approval have been obtained. If your research was exempt from ethical approval or permissions, please state so in your statement and provide a reason for exemption. Detailed guidance on ethics considerations and mandatory declarations can be found in our Editorial Policies section on Research Ethics (https://authorservices.taylorandfrancis.com/editorial-policies/research-ethics-and-consent/#ethicalconsiderations)."

☑ English checked by a professional editor or a colleague fluent in English before submission. [for authors whose first language is not English.]
